# The influence of team ethical leadership on ethical climate, moral efficacy, and multilevel vigilante behavior: the moderating role of organizational monitoring intensity

**DOI:** 10.3389/fpsyg.2026.1793533

**Published:** 2026-02-26

**Authors:** Zixin Zhang, Aimin Wang, Huili Ye

**Affiliations:** 1 School of Management, Wuhan University of Technology, Wuhan, China; 2 School of Management, Huazhong University of Science and Technology, Wuhan, China

**Keywords:** ethical climate, ethical leadership, moral efficacy, organizational vigilantism, social cognitive theory

## Abstract

**Introduction:**

Drawing on social cognitive theory, this study examines how team ethical leadership shapes organizational vigilantism across levels. We develop a dual-pathway, multilevel model linking team ethical leadership to vigilante behavior at both the team and individual employee levels, and propose organizational monitoring intensity as a key boundary condition.

**Methods:**

We tested hypotheses using a multi-wave field survey conducted in a large enterprise in Central China, including 92 teams and 303 employees. Multilevel analyses were used to assess cross-level mediation and moderated indirect effects.

**Results:**

Results showed that (1) team ethical leadership is positively associated with team vigilante behavior via team ethical climate; (2) team ethical leadership is positively associated with employee vigilante behavior via employee moral efficacy; and (3) organizational monitoring intensity negatively moderates these relationships. Specifically, ethical leadership more strongly predicts team ethical climate and employee moral efficacy—and their indirect effects on vigilantism at both levels—when monitoring intensity is low rather than high.

**Discussion:**

These findings indicate that ethical leadership functions as an important source of agency that encourages employees and teams to uphold norms, particularly when formal monitoring is weak. Practically, organizations may strengthen ethical order by prioritizing ethical leadership as a substitute for surveillance. The study advances vigilantism research by clarifying cross-level mechanisms and identifying monitoring intensity as a critical boundary condition.

## Introduction

In the digital economy era, the ethical challenges faced by enterprises are becoming increasingly complex and concealed. From the Facebook data breach to the certification flaws of Boeing 737 MAX, ethical failures in business have transcended traditional economic losses and evolved into systemic risks that threaten societal trust. According to the 2024 ACFE Global Fraud Report, over half of organizational whistleblowing cases originate from employees, and 32% of occupational fraud stems from inadequate internal controls (ACFE China, 2024). Against this backdrop, the question of how organizations can activate endogenous ethical enhancement mechanisms has become a shared concern for academia and business. Workplace vigilantes serve as monitors who intervene to discourage colleagues’ deviant or unethical behavior and to uphold shared ethical norms and standards. Organizational vigilantism refers to the self-initiated monitoring and intervention by employees within an organization. Employees, with their frequent interactions, possess unique observational advantages over formal oversight systems, enabling them to reduce blind spots in organizational supervision (DeCelles and Aquino, 2020). Furthermore, peer-to-peer informal monitoring and intervention are often more socially acceptable, making horizontal oversight a critical complement to vertical organizational control (Bitektine and Haack, 2015). Thus, vigilantism serves as a vital line of defense against ethical risks.

Although workplace vigilante behavior can function as an informal mechanism for discouraging misconduct, it is a self-appointed form of third-party enforcement that operates outside formal authority, understanding its antecedentsis theoretically central and practically urgent (DeCelles and Aquino, 2020). Precisely because vigilantism can shape whether and how norms are enforced in everyday work, a central theoretical task is to explain its antecedents—that is, what conditions trigger employees (and teams) to engage in vigilantism in the first place. However, extant research has not fully explained what systematically elicits vigilantism in organizational settings. A growing stream emphasizes who becomes a vigilante—especially identity-based accounts showing that individuals who internalize a “vigilante identity” are more motivated to monitor for norm violations and punish transgressors (Chen et al., 2022a). While valuable, this emphasis may under-specify organizational and leadership forces that can induce vigilantism across teams. In particular, leadership—one of the most powerful contextual determinants of employee ethical conduct—has received limited integration into models of vigilante behavior, despite strong evidence that ethical leadership shapes ethical norms, expectations, and behavioral standards (Brown and Treviño, 2006). This omission is consequential: without incorporating leadership and governance context, we cannot adequately explain when vigilantism is likely to emerge across teams, nor how organizations can intentionally shape conditions that trigger employee-led informal enforcement. Taken together, these gaps point to a need for an integrative, multilevel explanation that connects leadership cues in the work environment to the psychological and normative mechanisms through which vigilantism is activated. In other words, if vigilantism is an informal form of norm enforcement, then the key question becomes: what features of the ethical context signal that such enforcement is expected, legitimate, and feasible for employees to carry out?

**Figure 1:**
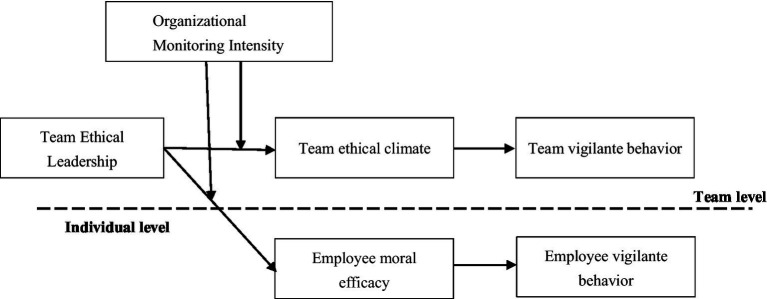
The research model.

To address this gap, we develop a cross-level antecedent model grounded in social cognitive theory, which conceptualizes behavior as the product of reciprocal influences between environmental cues and personal agency, with efficacy beliefs playing a pivotal role in translating moral standards into action (Bandura, 2001). This theoretical lens is particularly suitable because it clarifies how moral cues in the environment can (a) shape shared interpretations of “what we should do here” and (b) strengthen individuals’ confidence that they can act effectively when ethical issues arise—both of which are essential for initiating vigilantism. We propose that team ethical leadership acts as a salient moral cue that activates vigilantism through two complementary pathways. At the team level, ethical leadership cultivates a shared team ethical climate, which clarifies collective norms and legitimizes ethical intervention (Lu and Lin, 2014). At the individual level, ethical leadership enhances employees’ moral efficacy, strengthening their perceived capability to manage ethical issues and act on moral concerns (Lee et al., 2015). Importantly, we introduce organizational monitoring intensity as a boundary condition and argue—drawing on the substitutes for leadership perspective—that formal surveillance can function as a substitute for leadership influence (Kerr and Jermier, 1978). Accordingly, we predict that ethical leadership will be more strongly associated with ethical climate and moral efficacy—and thus more likely to elicit vigilante behavior—when monitoring intensity is low rather than high. Our research model is presented in Figure 1. By linking these team-level and individual-level mechanisms, our model explains not only who is motivated to engage in vigilantism, but also when vigilantism is likely to arise more broadly as a contextual response within teams.

This research makes four contributions. First, we shift the conversation from who becomes a vigilante to what elicits vigilante behavior, showing that vigilantism can be contextually induced rather than merely reflecting stable identities or dispositions (Chen et al., 2022b; DeCelles and Aquino, 2020). Second, integrating ethical leadership with social cognitive theory, we open the “black box” of how ethical leadership triggers vigilantism by identifying a dual-pathway mechanism: an environmental pathway via team ethical climate and an agency pathway via employees’ moral efficacy, demonstrating how leadership cues translate into informal enforcement at both team and individual levels (Bandura, 1991; Brown and Treviño, 2006). Third, by introducing organizational monitoring intensity as a boundary condition, we provide a contingency insight that formal monitoring can substitute for ethical leadership—ethical leadership is most influential when monitoring is low, whereas high monitoring attenuates leadership’s effects on ethical climate, moral efficacy, and the emergence of vigilantism (Kerr and Jermier, 1978).

## Hypothesis development

In the field of organizational behavior, ethical leadership is one of the most widely examined leadership styles. Ethical leaders are typically characterized as individuals with high moral standards; they not only demonstrate ethical traits and behaviors themselves but also engage in moral management and actively encourage employees to behave ethically in the workplace (Schaubroeck et al., 2012). As a result, ethical leadership could produce positive outcomes, such as reducing subordinates’ corrupt behaviors (Manara et al., 2020). In addition, Treviño et al. (2003) pointed out that ethical leadership is not only related to leaders’ personal characteristics (e.g., integrity and honesty) and ethical behaviors (e.g., fairness and ethical decision-making) but is also closely associated with management practices grounded in moral values, such as establishing and enforcing ethical standards.

Building on prior research on organizational climate, Victor and Cullen (1987) were the first to conceptualize ethical climate, defining it as a shared perception among organizational members regarding how ethical issues should be addressed and how ethical behavior should be identified and evaluated. Ethical climate is a specific type of organizational work climate, which can be understood as a normative climate shaped by organizational procedures, policies, and practices. When team members perceive that certain ethical standards or behavioral patterns are consistent with the expected norms guiding organizational decision-making, a team ethical climate emerges (Martin and Cullen, 2006). Importantly, team ethical climate is conceptually distinct from team ethical behavior: it captures shared perceptions and normative expectations about “what is appropriate” in the team, rather than the frequency of any specific ethical actions. Nevertheless, a stronger ethical climate is expected to foster ethical behavior and norm-supportive intervention by clarifying what the team collectively values and legitimizing members’ efforts to address misconduct when it occurs.

According to social cognitive theory, individuals are able to learn indirectly by observing the behaviors of others; through observation, imitation, and self-regulation, individuals internalize external environmental cues into personal behavioral standards (Bandura, 1999; Nabavi and Bijandi, 2012). Within a team, leaders are the most salient and influential figures. The behaviors of ethical leaders—such as transparent decision-making, open communication, and zero tolerance for unethical conduct—are collectively observed by team members. When team members observe that leaders’ ethical behaviors lead to positive outcomes (e.g., respect and trust), or that unethical behaviors are sanctioned within the organization. These shared observational experiences foster a convergent understanding of “what constitutes appropriate and accepted behavior within the team.” Over time, this process leads team members to develop similar shared cognitions. As Wimbush and Shepard (1994) noted, ethical climate reflects a consensus among organizational members regarding ethical conduct, and this shared understanding serves as a key reference for decision-making when ethical issues arise—forming the core of team ethical climate.

Therefore, drawing on social cognitive theory, this study proposes that ethical leadership transmits clear, consistent, and powerful ethical signals to the team through leaders’ personal moral behaviors (as collectively observed moral role models) and moral management practices (which shape the team’s ethical rules and reward–punishment environment). As team members jointly receive, interpret, and mutually validate these signals, they gradually develop shared perceptions of the team’s ethical policies, practices, and prevailing values, thereby forming a positive team ethical climate. In this way, ethical leadership shapes a shared normative context (i.e., ethical climate) that guides and energizes subsequent ethical behavior, including informal interventions to uphold team standards. Based on this reasoning, the following hypothesis is proposed:

Hypothesis 1: Team ethical leadership positively influences team ethical climate.

DeCelles and Aquino (2020) were the first to define “workplace vigilante behavior,” describing it as a self-appointed role in which an employee voluntarily assumes responsibility for monitoring and punishing coworkers’ deviant behaviors. By continuously monitoring the surrounding environment and sanctioning norm violators, workplace vigilantes can, on the one hand, effectively reduce deviant behavior and promote norm compliance, thereby facilitating effective organizational functioning. On the other hand, such informal monitoring and punishment may also expose vigilantes to criticism or social exclusion from coworkers. However, within teams characterized by a strong ethical climate—where members generally hold higher moral standards and share a collective understanding that ethical conduct is expected and valued—vigilante behavior is more likely to be perceived as legitimate, thereby reducing the likelihood of social exclusion.

According to social cognitive theory, human behavior is jointly determined by the interaction of cognitive factors, behavioral patterns, and the external environment; individuals are both shapers of, and shaped by, their environments. Drawing on this theoretical framework, the present study proposes that ethical leadership exerts a long-term influence on team-level vigilante behavior by using team ethical climate as a key transmission mechanism. Through the sustained reinforcement of team ethical climate, ethical leadership promotes team vigilante behavior for several reasons.

First, subordinates tend to feel psychologically safe when interacting with ethical leaders, which enhances their satisfaction with both leaders and work (Sharif and Scandura, 2014). In turn, followers are likely to reciprocate ethical leaders’ fairness and care by engaging in prosocial behaviors such as organizational citizenship behaviors (Kacmar et al., 2011). Legitimate vigilante behavior (e.g., punishing norm violators) can essentially be regarded as a form of prosocial behavior. Under a strong team ethical climate, team vigilante behavior is endowed with moral legitimacy: by reinforcing a sense of shared team responsibility and clarifying a collective attribution mechanism in which sanctioning responsibility is jointly borne by the team, ethical climate reduces individuals’ concerns about potential negative consequences. This, in turn, lowers tendencies toward responsibility avoidance and makes vigilante behavior more likely to be accepted and enacted by team members.

Second, ethical leaders shape team ethical climate by establishing moral standards, encouraging ethical behavior, and punishing unethical conduct, while simultaneously reinforcing the climate through consistent adherence to moral principles and alignment between words and deeds. Team ethical climate then provides members with a moral interpretive framework—explaining why vigilante behavior is appropriate—and enhances collective efficacy—members’ shared belief that the team is capable of successfully engaging in such behavior. As a result, team members are more likely to rationalize vigilante behavior, experience less cognitive dissonance, and develop stronger behavioral intentions to engage in vigilante actions. In short, the more salient and consistent the role-modeling behavior of ethical leaders, the stronger the shared ethical consensus formed through collective observation, the more positive the team ethical climate, and the more likely team members are to engage in vigilante behavior. This process reflects a “leadership shapes climate → climate promotes behavior” mechanism. Accordingly, the following hypothesis is proposed:

Hypothesis 2: Team ethical leadership has a positive influence on team vigilante behavior through the mediating role of team ethical climate.

In organizations, ethical leadership refers to management practices manifested through morally appropriate conduct, communication, and behavioral patterns. As role models, leaders are required to demonstrate high moral standards in their actions, thereby providing a moral framework for their followers (Demirtas, 2015). The reputation of ethical leadership is built upon two key foundations: leaders must be perceived not only as individuals with strong moral character, but also as leaders who actively engage in moral management (Treviño et al., 2000). Specifically, ethical leaders demonstrate normatively appropriate conduct through personal example and interpersonal interactions and convey these moral values to employees through two-way communication, institutional reinforcement, and participative decision-making processes (Brown et al., 2005).

According to social cognitive theory, self-efficacy is a central social-cognitive construct that significantly shapes individuals’ responses to risks and unexpected situations, as well as their confidence in creatively and effectively utilizing resources. Self-efficacy refers to individuals’ expectations and judgments regarding their capability to successfully perform a specific behavior in a given context (Bandura, 2013; Rullo et al., 2022). Moral efficacy, derived from the concept of self-efficacy, reflects individuals’ confidence in their ability to organize and mobilize internal motivations, external resources, and action strategies to behave ethically when facing moral dilemmas. Individuals with high moral efficacy are more likely to generate sufficient confidence and intrinsic motivation to engage in moral behavior under ethical challenges, whereas individuals with low moral efficacy may fail to translate moral judgments into moral actions (Hannah et al., 2011; Owens et al., 2019).

From a social cognitive perspective, individuals develop motivation through goal systems and are more willing to act—and believe they can succeed—when they anticipate positive outcomes and environmental support. Ethical leaders typically establish high moral standards, thereby providing followers with clear moral goals. Moreover, ethical leaders not only lead by example but also clearly communicate organizational support for ethical behavior and intolerance for unethical conduct through reward and punishment mechanisms. When subordinates observe leaders rewarding honesty and fairness while sanctioning deception and favoritism, they develop psychological expectations that ethical behavior will be recognized and protected within the organization. By fostering a fair and morally supportive organizational environment, ethical leadership reduces subordinates’ fears of potential negative consequences (e.g., social exclusion or loss of self-interest) associated with ethical actions, thereby strengthening their belief in their ability to successfully enact moral behavior.

In addition, ethical leaders engage in moral communication with followers by articulating ethical values and providing guidance when employees encounter moral ambiguity. When subordinates attempt to act ethically, the recognition, encouragement, or constructive feedback offered by ethical leaders directly reinforces the belief that “what I am doing is right, and my leader will support me.” This reinforcement enhances followers’ confidence in their capacity to confront ethical issues in the workplace and to persist in overcoming obstacles—an essential manifestation of moral efficacy. Accordingly, this study proposes the following hypothesis:

Hypothesis 3: Team ethical leadership positively influences employee moral efficacy.

One of the core concepts of social cognitive theory is self-efficacy (Bandura, 2001), defined as individuals’ beliefs in their ability to successfully perform specific behaviors. In moral contexts, this concept is extended to moral efficacy (Hannah et al., 2011), referring to individuals’ confidence in their ability to overcome obstacles and persist in moral action. Building on the core elements identified by DeCelles and Aquino (2020), Chen et al. (2022a) broadened the applicability of workplace vigilante behavior by redefining it as an individual’s self-perception that, even without formal authorization, one is willing to punish perceived norm violators when such action is considered appropriate and justified. Ethical leadership enhances this self-perception by strengthening employees’ moral efficacy, thereby motivating them to punish deviant behavior through informal means. The specific mechanisms are as follows:

First, team ethical leadership serves as an environmental input; through ethical behaviors such as fair treatment of employees and proactive responsibility-taking, ethical leaders provide observable role models that enhance employees’ beliefs in the feasibility and effectiveness of moral behavior. Moreover, by rewarding ethical conduct (e.g., public recognition) and sanctioning deviant behavior, ethical leaders signal organizational commitment to ethical standards, thereby increasing employees’ expectations that their own moral actions will be supported. Through role modeling and reinforcement mechanisms, ethical leadership thus enhances employees’ moral efficacy.

Moral efficacy constitutes a key psychological mechanism linking ethical leadership and employee vigilante behavior. According to social cognitive theory, behavior in moral situations depends on two primary expectations. The first is efficacy expectations—confidence in one’s ability to successfully execute moral behavior. Ethical leaders reduce employees’ perceived risks associated with moral action by establishing clear reward–punishment systems and protecting anonymous reporting channels, thereby strengthening confidence in engaging in legitimate vigilante behavior. The second is outcome expectations—evaluations of the likely consequences of one’s actions. By advocating and practicing principles of fairness, justice, and transparency, ethical leaders cultivate psychological safety, which reduces employees’ anxiety about potential failure or coworker retaliation and increases their willingness to engage in vigilante behavior. Taken together, ethical leadership enhances employees’ moral efficacy, and employees with high moral efficacy are more likely to initiate or persist in vigilante behavior to correct unethical conduct. Accordingly, the following hypothesis is proposed:

Hypothesis 4: Team ethical leadership positively influences employee vigilante behavior through the mediating role of employee moral efficacy.

Organizational monitoring refers to a supervisory approach in which organizations collect information about employees’ work progress and outcomes without directly requesting such information from subordinates. Typical practices include patrolling the workplace and observing employees’ task performance. This form of monitoring reflects a top-down management style (Liao and Chun, 2016). Organizational monitoring intensity refers to the extent to which organizations supervise and constrain employee behavior through formal systems, technologies, or procedures. Prior research has demonstrated the double-edged nature of organizational monitoring. On the one hand, monitoring provides senior managers with guidance for formulating and adjusting organizational strategies (Komaki et al., 1986). On the other hand, with advances in computer technology and the increasing prevalence of remote work, organizational monitoring has become more covert, intensive, and intrusive through relatively low-cost technological means (Holland et al., 2015). In this context, the psychological impact of organizational monitoring on employees has often been overlooked. Integrating prior research on electronic monitoring, Ravid et al. (2023) called for future studies to pay greater attention to employees’ psychological experiences under electronic organizational monitoring. Taken together, clarifying the mechanisms through which organizational monitoring operates is of critical importance for both scholars and practitioners.

Social cognitive theory (Bandura, 1999) emphasizes the triadic reciprocal interaction among individuals, behavior, and the environment. In organizational settings, leadership behavior (individual factors), team cognition (behavioral factors), and organizational control systems (environmental factors) form a dynamic interactive system. Although this study argues that ethical leadership, as an important environmental factor, facilitates the emergence of team ethical climate (H1), ethical leadership is not the only relevant environmental influence in organizations. Organizational monitoring, as another salient environmental factor, may strengthen or weaken the effect of ethical leadership on team ethical climate. Accordingly, this study treats organizational monitoring intensity as a boundary condition and examines how it moderates the relationship between ethical leadership and team ethical climate.

When organizational monitoring intensity is excessively high—such as when employees’ entire work processes are observed or their movements and task progress are tracked whenever and wherever possible. Employees may feel distrusted, and experience heightened psychological strain, triggering psychological reactance (Niehoff and Moorman, 1993). Under such conditions, organizational monitoring may function as an ethical constraint and foster ‘instrumental ethical cognition’, whereby employees comply with ethical norms primarily to avoid punishment rather than because they genuinely endorse ethical values. As a result, team members may attribute ethical compliance to external pressure rather than to the intrinsic influence of ethical leadership, which is detrimental to the development of a positive team ethical climate. In contrast, when organizational monitoring intensity is relatively low, teams lack clear institutional guidance and rely more heavily on leaders as primary sources of social information. Under conditions of ambiguous environmental cues and concentrated cognitive resources, team members devote greater attention to interpreting leaders’ behavioral patterns, thereby deepening observational learning (Daft and Weick, 1984). As a result, Team members are more likely to observe and learn the thoughts and behaviors of ethical leadership under low-intensity organizational monitoring, thereby fostering a positive ethical climate. Accordingly, this study proposes the following hypothesis:

Hypothesis 5: Organizational monitoring intensity moderates the positive relationship between team ethical leadership and team ethical climate, such that the relationship is stronger when organizational monitoring intensity is low rather than high.

Building on Hypothesis 5, this study further proposes a moderated mediation model in which team ethical climate mediates the effect of ethical leadership on team vigilante behavior, and the magnitude of this indirect effect is contingent on organizational monitoring intensity.

Based on the earlier theoretical development (H2), ethical leadership promotes team vigilante behavior by fostering a strong ethical climate, leading by implementing moral practices (e.g., protecting whistleblowers), thereby establishing a shared understanding that “maintaining ethics is a collective responsibility.” Prior research has shown that friendship-oriented ethical climates strengthen employees’ affective identification with the organization, increase organizational citizenship behaviors (OCBs), and reduce counterproductive work behaviors (CWBs) (Pagliaro et al., 2018). Vigilante behavior—including, but not limited to, proactively reporting misconduct—represents a typical form of organizational citizenship behavior. However, in high-intensity monitoring environments, formal surveillance systems convey an implicit signal that “the organization does not trust employees to self-regulate.” This signal contradicts the fair and reasoned organizational environment promoted by ethical leadership and the emphasis on autonomous moral agency inherent in a positive team ethical climate. Such inconsistency blurs normative reference points and may create cognitive conflict among team members, thereby reducing the likelihood of action and suppressing vigilante behavior.

By contrast, in low-intensity monitoring environments, ethical leaders must rely more heavily on frequent moral actions—such as proactively adhering to ethical standards and openly discussing ethical issues—to compensate for institutional gaps. According to social cognitive theory, individuals learn indirectly by observing others’ behavior. Team members are therefore more likely to observe and emulate ethical leaders’ moral actions, creating a positive feedback loop of observation and learning. Moreover, under conditions of loose organizational monitoring, team ethical climate is more likely to serve as a key interpretive framework for employee behavior. In such contexts, vigilante behavior is more readily attributed to internal, value-driven motivations, increasing team members’ willingness to engage in vigilante actions.

Hypothesis 6: Organizational monitoring intensity moderates the indirect effect of team ethical leadership on team vigilante behavior via team ethical climate, such that the indirect effect is stronger when organizational monitoring intensity is low rather than high.

Drawing on social cognitive theory (Bandura, 1986), ethical leadership influences a key mechanism underlying the development of efficacy beliefs—vicarious experience, defined as observing others successfully perform a task. Vicarious experience plays a critical role in shaping followers’ efficacy beliefs. As close observers of ethical leaders in their roles as moral agents, followers are often involved—albeit indirectly—in leaders’ processes of addressing complex moral dilemmas. When ethical leaders confront difficult ethical challenges in a careful, comprehensive, and exploratory manner, followers are able to observe which strategies are effective and which are not. Through such vicarious experiences, followers gain confidence in their own ability to cope with future moral dilemmas, thereby enhancing their moral efficacy. This reasoning forms the basis of Hypothesis 3, which proposes that ethical leadership promotes the development of followers’ moral efficacy. However, individuals’ moral efficacy is also shaped by other environmental factors, such as business ethics education (May et al., 2014). Accordingly, the present study focuses on an important contextual factor—organizational monitoring—and examines how it influences the effect of ethical leadership on followers’ moral efficacy.

Organizational monitoring is widespread across industries. In a study of U. S. police departments, Adams and Mastracci (2019) found that the widespread adoption of body-worn cameras produced unintended negative consequences. Because these devices continuously record and transmit officers’ work activities, such high intensity monitoring not only weakened officers’ sense of organizational belonging but also significantly increased job burnout. High-technology monitoring thus exhibits a clear double-edged effect in organizational management. On the one hand, continuous surveillance may induce negative outcomes such as employee burnout; on the other hand, its protective and safety-enhancing value cannot be ignored. For example, Robson et al. (2016) found that fatigue-sensing headbands worn by heavy machinery operators effectively reduced workplace accidents. Addressing this paradox, Bhave (2014) conducted a meta-analysis and demonstrated that although organizational monitoring improves employee efficiency in standardized processes, it undermines performance on complex tasks requiring creativity. Taken together, organizational monitoring exerts multidimensional effects on individuals.

From a social cognitive perspective, ethical leadership constructs moral behavior templates for organizational members through symbolic modeling. When leaders are perceived as credible moral role models, this role-modeling process activates observational learning. When employees perceive leaders’ moral demonstrations as developmental support rather than evaluative threat, moral efficacy is positively reinforced (Rullo et al., 2022). Notably, low organizational monitoring intensity functions as a contextual reinforcing condition that is structurally aligned with the procedurally fair environment promoted by ethical leadership. This dual influence enables employees to perceive clear responsibility boundaries while retaining autonomy, thereby continuously enhancing followers’ moral efficacy. In contrast, under conditions of high organizational monitoring intensity, employees’ attention tends to be fixated on compliance indicators (e.g., whether non-work-related websites are accessed during work hours), which reduces selective attention to leaders’ moral modeling. As a result, leaders’ role-modeling effects are diluted, observational learning is weakened, and the development of employees’ moral efficacy is hindered. Based on this reasoning, the following hypothesis is proposed:

Hypothesis 7: Organizational monitoring intensity moderates the positive relationship between team ethical leadership and employee moral efficacy, such that the relationship is stronger when organizational monitoring intensity is low rather than high.

Prior literature has documented the positive effects of ethical leadership on followers from multiple theoretical perspectives. Drawing on social learning theory, Zhang et al. (2018) demonstrated that ethical leadership promotes followers’ moral behaviors by activating moral emotions, as reflected in increased willingness to report unethical conduct and reduced misconduct. From a social cognitive perspective, Wu (2021) further found that ethical leadership fosters a positive organizational moral ecology by strengthening organizational members’ group identification, thereby facilitating constructive behaviors such as knowledge sharing. These findings align with Hypothesis 4 of the present study, which proposes that ethical leadership positively predicts employee vigilante behavior through enhanced moral efficacy. In addition, Hypothesis 7 identifies organizational monitoring intensity as a moderator of the relationship between ethical leadership and employee moral efficacy. Integrating Hypotheses 4 and 7, we propose a moderated mediation model with the following mechanism.

When organizational monitoring intensity is high, external control systems based on strict rules and reward–punishment mechanisms substantially reduce employees’ need for self-regulation. Under such conditions, the pathway through which ethical leadership influences behavior via moral efficacy is substituted by external control mechanisms, thereby weakening the mediating effect. In contrast, under conditions of low organizational monitoring intensity, the absence of strong external constraints leads employees to rely more heavily on internalized moral cognitive systems when making behavioral decisions. This allows the modeling effects of ethical leadership to be more readily absorbed and internalized, strengthening moral efficacy as an intrinsic motivational force and enhancing its explanatory power for vigilante behavior. Accordingly, the following hypothesis is proposed:

Hypothesis 8: Organizational monitoring intensity moderates the indirect effect of team ethical leadership on employee vigilante behavior via employee moral efficacy, such that the indirect effect is stronger when organizational monitoring intensity is low rather than high.

## Method

### Participants and procedure

Data were collected in a large pharmaceutical enterprise located in Hubei Province, China, using a multi-source, time-lagged survey design. For recruitment, the research team first established contact with the company’s CEO and obtained organizational support for the study. The human resources (HR) department then provided a roster of eligible team leaders and team members who participated in the survey administration. Regarding sampling, our approach was best characterized as a total-enumeration strategy within the focal organization: we attempted to invite all eligible teams listed in the HR roster during the data-collection period. Eligibility was defined ex ante based on organizational records—specifically, teams with a formally designated team leader and stable leader–member reporting relationships to ensure adequate exposure to leadership behaviors and team norms. HR’s roster served as the sampling frame, and all teams meeting these criteria were invited to participate across waves; thus, the final sample reflects participation and successful matching rather than selective recruitment of a subset of teams.

All procedures involving human participants followed the ethical standards of the Helsinki Declaration. All participants were informed of the academic purpose of the study, assured that participation was voluntary and confidential, and provided informed consent before responding. Given the potentially sensitive nature of workplace vigilantism, we implemented additional safeguards to reduce any perceived risk or discomfort. Specifically, participants were explicitly informed that (a) the survey was for academic research only, (b) their individual responses would not be disclosed to the organization, supervisors, or HR, and (c) they could skip any question or withdraw at any time without penalty. To reduce evaluation apprehension, we emphasized that there were no “right” or “wrong” answers and encouraged honest responding. No direct personal identifiers (e.g., names or employee ID numbers) were collected in the questionnaires. All survey data were stored, and access was restricted to the research team.

To reduce potential common method bias and to improve temporal separation among key constructs, questionnaires were administered to team leaders and their direct team members at different time points. Respondents completed the surveys voluntarily and were instructed to answer independently. To enable leader–employee matching across waves while preserving anonymity, each work team was assigned an identification code that was used consistently across all survey rounds. These identification codes were generated by the research team solely for matching purposes and did not correspond to any company-internal identifiers. HR’s role was limited to distributing sealed envelopes containing the coded surveys to participants; neither HR nor organizational leaders had access to raw data. During analysis, matching was performed using identification codes only, and the dataset contained no information that could be used to identify individual respondents.

The study comprised three measurement waves. At Time 1 (T1), questionnaires were distributed to 143 team leaders to assess team ethical leadership, organizational monitoring intensity, and leaders’ demographic information; 139 leader surveys were returned. At Time 2 (T2), leaders reported team ethical climate, while employees reported employee moral efficacy and provided demographic information; usable data were obtained from 98 teams and 320 employees. At Time 3 (T3), leaders rated team vigilante behavior, and employees rated employee vigilante behavior; responses were collected from 95 teams and 313 employees. After removing cases that could not be matched across waves and/or contained incomplete or inconsistent identifiers, the final matched sample consisted of 92 teams, including 92 team leaders and 303 employees.

In the final employee sample (*N* = 303), 39.604% were men and 60.396% were women. Employees’ age distribution was 5.941% aged 21–30, 34.983% aged 31–40, 48.845% aged 41–50, and 10.231% aged 51–60. Regarding tenure, 35.644% had worked for <3 years, 29.043% for 3–5 years, 19.472% for 5–10 years, and 15.842% for >10 years. For education, 8.911% had a high school degree or below, 30.033% held an associate degree, 60.396% held a bachelor’s degree, 0.330% held a master’s degree, and 0.330% held a doctoral degree. In the leader sample (N = 92), 40.217% were men and 59.783% were women. Leaders’ age distribution was 1.087% aged 21–30, 23.913% aged 31–40, 63.043% aged 41–50, and 11.957% aged 51–60. Leaders’ tenure was 9.783% < 3 years, 22.826% 3–5 years, 33.696% 5–10 years, and 33.696% > 10 years. For education, 9.783% had a high school degree or below, 44.565% held an associate degree, and 45.652% held a bachelor’s degree.

### Measures

All focal constructs were assessed using multi-item scales on **seven-point Likert-type response formats**. Unless otherwise specified, response options ranged from **1 = strongly disagree** to **7 = strongly agree**. For leader-rated variables, team leaders provided evaluations based on their perceptions of their teams and their own behaviors. For employee-rated variables, employees reported their perceptions or self-assessments. Items were translated into Chinese using a standard translation and back-translation procedure to ensure semantic equivalence.

#### Team ethical leadership (leader-rated; T1)

Team ethical leadership was measured with the 10-item scale developed by Schaubroeck et al. (2016). Team leaders evaluated the extent to which they demonstrated ethical leadership behaviors in managing their teams. A sample item is: “Sets an example of how to do things the right way in terms of ethics.” The internal consistency reliability for this scale was **Cronbach’s *α* = 0.933**.

#### Organizational monitoring intensity (leader-rated; T1)

Organizational monitoring intensity was assessed with a 3-item measure adapted from Wells et al. (2007). Leaders reported the extent to which the organization closely monitors employees’ behaviors, particularly with respect to preventing misconduct and deviations from organizational norms. A sample item is: “The organization closely monitors employees to prevent wrongdoing on the part of employees.” The internal consistency reliability for this scale was **Cronbach’s *α* = 0.908**.

#### Team ethical climate (leader-rated; T2)

Team ethical climate was measured using a 13-item scale drawn from Cheng and Wang (2015). Team leaders rated the ethical norms and expectations that characterize their teams. A sample item is: “In this team, people are expected to follow their own personal and moral beliefs.” The internal consistency reliability for this scale was **Cronbach’s *α* = 0.839**.

#### Employee moral efficacy (employee-rated; T2)

Employee moral efficacy was assessed with the 9-item scale developed by May et al. (2014). Employees indicated their confidence in their ability to manage ethical issues at work. Following the original scale, responses were anchored from 1 = Not Confident at All to 7 = Very Confident. A sample item is: “Making suggestions to management about ways to improve the working of your section concerning ethical issues.” The internal consistency reliability for this scale was **Cronbach’s *α* = 0.950**.

#### Team vigilante behavior (leader-rated; T3)

Team vigilante behavior was measured with a 10-item scale adapted from Chen et al. (2022b). Leaders evaluated the extent to which team members collectively engaged in informal punitive or accountability-seeking behaviors toward perceived wrongdoing. Items were introduced with a stem indicating the target referent (e.g., “In our team, members…”). A sample item is: “Makes sure people are held accountable when they do something wrong.” The internal consistency reliability for this scale was **Cronbach’s α = 0.867**.

#### Employee vigilante behavior (employee-rated; T3)

Employee vigilante behavior was measured with the same 10-item adapted scale based on Chen et al. (2022b), but framed at the individual level. Employees reported the extent to which they personally engaged in vigilante-type behaviors at work. The item wording was adjusted to a self-referent format while retaining the original meaning. A sample item is: “I make sure people are held accountable when they do something wrong.” The internal consistency reliability for this scale was **Cronbach’s α = 0.901**.

#### Control variables

Consistent with prior research on leadership and team processes, we controlled for leaders’ demographic characteristics, including leader gender, education level, and leader organizational tenure, because these factors may be associated with leaders’ perceptions and behavioral tendencies as well as team functioning.

### Analytical strategy

Given the nested structure of the data (employees nested within teams) and the conceptual distinction between team-level and individual-level processes (see Figure 1). We specified TYPE = TWOLEVEL models to simultaneously estimate within-team (Level 1) and between-team (Level 2) relations, thereby accounting for the non-independence of observations and avoiding biased standard errors that may arise from single-level analyses.

In line with the research model, team ethical leadership and organizational monitoring intensity were modeled at the between-team level. Team ethical climate and team vigilante behavior were also modeled as team-level constructs. At the within-team level, employee moral efficacy and employee vigilante behavior were modeled as employee-level variables. Cross-level pathways were estimated where theoretically appropriate (e.g., team ethical leadership predicting employee moral efficacy), and indirect effects were tested to evaluate the proposed multilevel mediation mechanisms linking leadership to vigilante behavior through ethical climate and moral efficacy. All paths were estimated simultaneously in a single multilevel path model.

## Results

### Confirmatory factor analyses

To establish the discriminant validity of the focal constructs, we conducted a series of confirmatory factor analyses (CFAs) using Mplus. Given the multilevel structure of the data (employees nested within teams), CFAs were specified consistent with a two-level framework, such that employee-rated constructs were modeled at the within-team level and leader-rated team constructs were modeled at the between-team level.

Model fit was evaluated using multiple indices, including the chi-square statistic (χ^2^), the comparative fit index (CFI), the Tucker–Lewis’s index (TLI), the root-mean-square error of approximation (RMSEA), and the standardized root-mean-square residual (SRMR; within and between). The results showed in Table 1, the hypothesized six-factor model demonstrated better fit to the data (χ^2^ = 102.212, df = 56, CFI = 0.971, TLI = 0.958, RMSEA = 0.052, SRMR_within = 0.028, SRMR_between = 0.050). And those alternative models fit the data significantly worse than the hypothesized model, providing support for the distinctiveness of the study variables.

**Table 1:** Confirmatory Factor Analyses (CFA) Results and Model Comparisons.

Model	Factor structure	χ^2^	df	CFI	TLI	RMSEA	SRMR_W	SRMR_B
Model 1 (Hypothesized)	6-factor: TEL, OMI, TEC, EME, TVB, EVB	102.212	56	0.971	0.958	0.052	0.028	0.050
Model 2	5-factor: combine TEC and TVB	137.482	59	0.951	0.933	0.066	0.028	0.083
Model 3	5-factor: combine EME and EVB	559.694	57	0.686	0.554	0.171	0.211	0.050
Model 4	4-factor: combine (TEC + TVB), (EME + EVB)	572.479	60	0.680	0.568	0.168	0.211	0.083
Model 5	2-factor: combine (TEC + TVB + OMI), (EME + EVB)	1009.640	63	0.409	0.240	0.223	0.211	0.192

Fit indices are reported for multilevel CFA in Mplus. TEL = team ethical leadership; OMI = organizational monitoring intensity; TEC = team ethical climate; EME = employee moral efficacy; TVB = team vigilante behavior; EVB = employee vigilante behavior.

### Descriptive statistics and correlations

Table 2 presents the means, standard deviations, and zero-order correlations among the study variables. At the team level, team ethical leadership was positively associated with team ethical climate (*r* = 0.467, *p* < 0.01) and positively associated with team vigilante behavior (*r* = 0.361, *p* < 0.01). Organizational monitoring intensity was positively related to team ethical climate (*r* = 0.315, *p* < 0.01). At the individual level, employee moral efficacy was positively related to employee vigilante behavior (*r* = 0.214, *p* < 0.01). Overall, the correlation pattern was broadly consistent with the proposed model and provided preliminary support for the hypothesized relationships.

**Table 2:** Means, standard deviations, reliabilities, and correlations.

Variables	Mean	SD	1	2	3	4	5	6	7	8	9	10
Between level												
1. Leader gender	1.588	0.493	1									
2. Leader age	3.850	0.602	0.143^*^	1								
3. Leader tenure	2.970	0.983	−0.111	0.167^**^	1							
4. Leader education level	2.350	0.637	0.151^**^	0.226^**^	−0.355^**^	1						
5. Ethical leadership	6.346	0.694	0.014	−0.170^**^	0.081	−0.169^**^	1					
6. Organizational monitoring intensity	6.182	0.998	−0.065	−0.006	−0.049	0.08	0.167^**^	1				
7. Team ethical climate	6.190	0.543	0.039	−0.168^**^	−0.202^**^	−0.032	0.467^**^	0.315^**^	1			
8. Team vigilante behavior	6.088	0.709	0.025	−0.166^**^	−0.220^**^	0.094	0.361^**^	0.386^**^	0.538^**^	1		
Within level												
9. Employee moral efficacy	5.066	1.172	−0.003	−0.079	0.03	−0.138^*^	0.106	0.045	0.229^**^	0.125^*^	1	
10. Employee vigilante behavior	4.760	1.159	0.008	0.069	0.124^*^	−0.102	0.051	0.084	−0.043	0.067	0.214^**^	1

*N*_team_ = 92, *N*_employee_ = 303. ^*^p < 0.05, ^**^p < 0.01.

### Hypothesis testing

Hypotheses were tested using multilevel structural equation modeling in Mplus (TYPE = TWOLEVEL), which simultaneously estimated relationships at the team (between) and individual (within) levels. Leader gender, leader education, and leader tenure were included as control variables at the between-level.

As shown in Table 3, team ethical leadership was positively associated with team ethical climate (*γ* = 0.472, *p* < 0.01), supporting **Hypothesis 1**. Team ethical leadership also positively predicted employee moral efficacy (*γ* = 0.305, *p* < 0.05), supporting **Hypothesis 3**. Consistent with Hypothesis 2, team ethical climate was positively related to team vigilante behavior (*γ* = 0.374, *p* < 0.01), and the indirect effect of team ethical leadership on team vigilante behavior via team ethical climate was significant (indirect effect = 0.175, 95% CI [0.061, 0.290]). In addition, consistent with **Hypothesis 4**, employee moral efficacy was positively related to employee vigilante behavior (*β* = 0.223, *p* < 0.01). The indirect effect of team ethical leadership on employee vigilante behavior through employee moral efficacy was positive and marginally significant (indirect effect = 0.058). Importantly, this indirect effect was significant at the 90% confidence interval (90% CI [0.003, 0.114]), although the 95% confidence interval marginally included zero (95% CI [−0.008, 0.124]). Taken together, these findings provide support for the proposed team-level and individual-level mediating mechanisms.

**Table 3:** Multilevel Regression Results.

Variables	Team ethical climate	Employee moral efficacy	Employee vigilante behavior	Team vigilante behavior
	Model 1	Model 2	Model 3	Model 4
Controls				
Leader gender	−0.014			−0.042
Leader education	−0.012			0.129
Leader tenure	−0.223^**^			−0.137^+^
Cross-level predictors (Between-level)				
Team ethical leadership	0.472^**^	0.305^*^	0.188	0.152^+^
Organizational monitoring intensity	0.275^**^	0.135	0.414	0.179^+^
TEL × OMI	−0.255^**^	−0.514^**^		
Team ethical climate				0.374^**^
Within-level				
Employee moral efficacy			0.223^**^	
*R* ^2^	0.388	0.327	0.287	0.359

*N*_team_ = 92, *N*_employee_ = 303. +*p* < 0.1, ^*^p < 0.05, ^**^p < 0.01.

**Figure 2:**
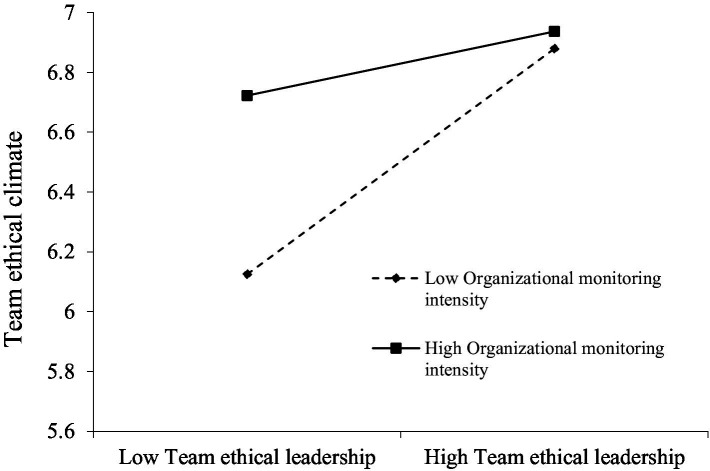
Organizational monitoring intensity as a moderator of the relationship between team ethical leadership and team ethical climate.

To test moderation, we created an interaction term between team ethical leadership and organizational monitoring intensity at the between-team level and examined its effects on the mediators. Results indicated that the interaction significantly predicted team ethical climate (*γ* = −0.255, *p* < 0.01), supporting **Hypothesis 5**. As shown in Figure 2, simple-slope analyses revealed that the positive association between team ethical leadership and team ethical climate was stronger when organizational monitoring intensity was low (simple slope = 0.527, *p* < 0.01) than when it was high (simple slope = 0.170, *p* < 0.01). These patterns suggest that formal monitoring partially “crowds out” the normative and sense giving role of ethical leadership: when monitoring systems are strong, teams may rely less on leaders’ ethical cues to form shared perceptions about “how we do things here,” resulting in a weaker leadership–climate linkage. Conversely, when monitoring is weak, ethical leadership becomes a more central source of guidance for defining and reinforcing ethical expectations, thereby exerting a stronger effect on the team’s ethical climate.

**Figure 3:**
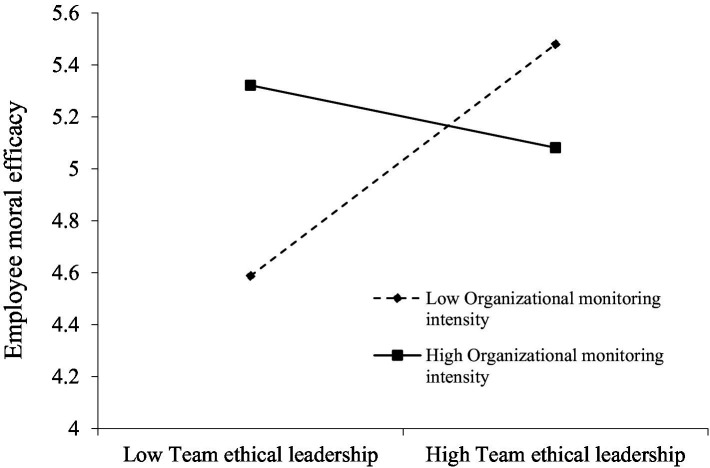
Organizational monitoring intensity as a moderator of the relationship between team ethical leadership and employee moral efficacy.

Similarly, the interaction between team ethical leadership and organizational monitoring intensity significantly predicted employee moral efficacy (*γ* = −0.514, *p* < 0.01), supporting **Hypothesis 7.** As Figure 3 shows, simple-slope tests indicated that team ethical leadership had a stronger positive relationship with employee moral efficacy under low organizational monitoring intensity (simple slope = 0.610, *p* < 0.01), whereas the relationship was nonsignificant when monitoring intensity was high (simple slope = −0.140, n.s.). This interaction implies that ethical leadership is most effective in building employees’ confidence to handle ethical issues when formal oversight is limited, because employees may need to exercise greater personal discretion and thus draw more heavily on leaders’ modeling and encouragement. In contrast, under high monitoring intensity, compliance-oriented surveillance may shift employees’ focus on rule-following and external control, leaving less room for leader-driven efficacy-building processes.

As shown in Table 4, we further examined conditional indirect effects to test moderated mediation. In support of **Hypothesis 6**, the indirect effect of team ethical leadership on team vigilante behavior via team ethical climate was stronger when organizational monitoring intensity was low (indirect effect = 0.265, 95% CI [0.073, 0.457]) than when it was high (indirect effect = 0.086, 95% CI [0.024, 0.147]). The difference between these two indirect effects was significant (difference = 0.180, 95% CI [0.009, 0.350]).

**Table 4:** Simple indirect effects and conditional indirect effects.

Hypothesis	Indirect path	Indirect effect	SE	95% CI	90% CI
H2	TEL → TEC → Team vigilante behavior	0.175	0.059	[0.061, 0.290]	[0.079, 0.272]
H4	TEL → EME → Employee vigilante behavior	0.058	0.034	[−0.008, 0.124]	[0.003, 0.114]

Hypothesis	Conditional indirect path	Low OMI (−1 SD) Indirect; 95% CI	High OMI (+1 SD) Indirect; 95% CI	Difference; 95% CI
H6	TEL → TEC → Team Vigilante Behavior	0.265 [0.073, 0.457]	0.086 [0.024, 0.147]	0.180 [0.009, 0.350]
H8	TEL → EME → Employee Vigilante Behavior	0.151 [0.023, 0.280]	−0.035 [−0.110, 0.041]	0.186 [0.022, 0.351]

*N*_team_ = 92, *N*_employee_ = 303. Indirect effects are products of coefficients (a × b). ^*^p < 0.05, ^**^p < 0.01.

Likewise, consistent with **Hypothesis 8**, the indirect effect of team ethical leadership on employee vigilante behavior via employee moral efficacy was stronger at low organizational monitoring intensity (indirect effect = 0.151, 95% CI [0.023, 0.280]) than at high monitoring intensity (indirect effect = −0.035, 95% CI [−0.110, 0.041]). The difference between these two indirect effects was significant (difference = 0.186, 95% CI [0.022, 0.351]).

Taken together, these findings suggest that organizational monitoring intensity attenuates the positive effects of ethical leadership on ethical climate and moral efficacy, thereby weakening the indirect effects on vigilante behavior.

## Discussion

This study aimed to investigate how and when ethical leadership influences organizational vigilantism across different levels. Based on social cognitive theory, we developed a multilevel model and tested it using leader-follower matched data from a large Chinese enterprise. Our findings confirm that ethical leadership significantly promotes organizational vigilante behavior. Specifically, this relationship is mediated by team ethical climate at the team level and by employee moral efficacy at the individual level. Furthermore, we identified organizational monitoring intensity as a critical boundary condition. In the following sections, we discuss the theoretical contributions, practical implications, and limitations of this study.

### Theoretical implications

Our study offers three distinct contributions to the literature on organizational deviance and leadership. First, we expand the scope of deviance management from macro-level formal intervention to micro-level interpersonal control by establishing the significance of organizational vigilante behavior. Traditionally, scholars have focused on how organizations suppress deviance through vertical, top-down powers, such as formal regulations and sanctions (Kidwell and Martin, 2004). In contrast, limited attention has been paid to the horizontal power dynamics where employees proactively police one another. Although DeCelles and Aquino (2020) theoretically proposed that workplace vigilantism serves as a vital supplement to formal authority, empirical research on this construct remains scarce in management literature, having been traditionally confined to criminology and political science (Weisburd, 1988; Rosenbaum and Sederberg, 1976). By empirically validating the antecedents and mechanisms of team and employee vigilante behavior, our study answers the call to explore how frontline employees exercise agency to reconstruct work orders (Piazza et al., 2024). We position vigilantism as a critical form of micro-social control, suggesting that effective deviance management relies not only on management-led heteronomy but also on the activation of employee-led co-governance.

Second, we shift the research focus from “*who becomes a vigilante*” (i.e., traits) to “*when and how vigilantism is induced*” (i.e., context), breaking through the “trait-based” paradigm of antecedent research by identifying ethical leadership as a key situational inducer, responding to calls for research on the contextual antecedents of vigilantism. Existing research has predominantly focused on the dispositional antecedents of vigilantism, examining individual differences such as personality traits (Moisuc et al., 2018), individualism–collectivism orientations (Wang et al., 2023), or the possession of a specific vigilante identity derived from victimhood (Chen et al., 2022a; Chen et al., 2022b). These studies largely treat vigilantism as a stable expression of the self, potentially overlooking the role of organizational context. Our study fills this gap by demonstrating that vigilantism is not merely an innate tendency of a few “Dark Knights” but a behavior that can be actively cultivated by ethical leaders. By proving that ethical leadership serves as a situational catalyst, we broaden the understanding of vigilantism from a trait-based phenomenon to a malleable, leadership-driven process, significantly expanding the intervention pathways for vigilantism, proving that organizations can proactively shape employees’ social enforcement behaviors by optimizing leadership styles rather than passively waiting for individual traits to manifest.

Third, drawing on social cognitive theory, we unpack the “black box” of the leadership-vigilantism relationship by revealing a dual-pathway mechanism. While recent studies have explored the cognitive underpinnings of vigilantism, they have mostly focused on self-verification processes (Chen et al., 2022a) or the negative consequences on job outcomes (Iqbal et al., 2022). The generative mechanism explaining how external leadership signals translate into specific enforcement behaviors has remained unclear. We advance this line of inquiry by uncovering two distinct pathways: an environmental pathway via *team ethical climate* and a cognitive pathway via *employee moral efficacy*. We show that ethical leaders do not merely model behavior; they construct a supportive moral environment that legitimizes intervention and simultaneously boosts employees’ internal confidence to act. This confirms the social cognitive view that vigilantism is a product of the dynamic interaction between environmental cues and personal agency.

Fourth, we clarify the boundary conditions of informal control by revealing the “substitution effect” between ethical leadership and organizational surveillance. DeCelles and Aquino (2020) theorized that vigilantes often emerge to fill a “control void” where formal authority is absent, yet empirical tests of the interaction between formal and informal controls have been lacking. Our study offers new insights by integrating the “Substitutes for Leadership” framework (Kerr and Jermier, 1978). We find that ethical leadership (i.e., informal control) and organizational monitoring intensity (i.e., formal control) function as substitutes rather than complements. Specifically, the positive impact of ethical leadership on team and employee vigilante behavior is most potent when organizational monitoring intensity is low. Conversely, high-intensity organizational monitoring tends to crowd out the motivational effect of leadership. This finding refines the contingency perspective of social control, highlighting that soft leadership is most critical for maintaining order when hard systemic monitoring is weak.

### Practical implications

First, organizations should prioritize the cultivation of ethical leadership to stimulate proactive vigilante behaviors. Since ethical leadership serves as a crucial antecedent for both team and individual organizational vigilantism, companies should move beyond merely selecting leaders based on technical competence. Instead, recruitment and promotion criteria should explicitly include moral character and ethical track records. For example, organizations can incorporate structured behavioral interviews, ethics-focused assessment centers, and multi-source reference checks that explicitly evaluate candidates’ integrity and fairness. Furthermore, leadership training programs should be designed to enhance leaders’ capabilities in role modeling and two-way communication regarding ethics. To make training actionable, firms can require leaders to practice and receive feedback on how to respond to reported misconduct (e.g., listening, documenting, protecting confidentiality, and escalating appropriately). In addition, organizations can institutionalize ethical leadership through performance management by including ethics-related KPIs (e.g., frequency/quality of ethics conversations, employee-rated fairness) and linking them to rewards and promotion decisions. By doing so, leaders can effectively signal that upholding organizational norms is a valued behavior, thereby encouraging employees to act as informal guardians, vigilantes, who actively maintain workplace ethical order.

Second, managers should actively foster a supportive team ethical climate, and boost employees’ moral efficacy to facilitate the translation of leadership into action. Our findings indicate that ethical leadership influences vigilante behavior through dual pathways: the collective environment, i.e., climate, and individual confidence, i.e., moral efficacy. Therefore, organizations should encourage open dialogues about ethical dilemmas to build a shared perception of “right vs. wrong” within teams. To operationalize this, managers can implement structured ethical voice routines, such as short, anonymized dilemma polls to identify recurring gray areas that require clarification. Simultaneously, managers should focus on empowering employees by providing positive feedback and recognizing their moral courage. Organizations can strengthen moral efficacy by offering micro-skill training, for example, how to confront respectfully, how to document facts, and how to seek allies, providing templates for reporting and escalation, and clarifying what constitutes appropriate versus inappropriate intervention to prevent overreach. Strengthening employees’ belief in their own moral capabilities ensures they possess the psychological resources necessary to intervene when they witness deviance, rather than remaining passive bystanders. Importantly, firms can establish “safe intervention channels” (e.g., ombudspersons, confidential hotlines) so employees have multiple pathways to act without fear of retaliation. To avoid vigilantism becoming harmful or retaliatory, organizations should pair encouragement of intervention with clear guardrails—explicit anti-retaliation policies, training on proportional responses, and guidance emphasizing constructive actions rather than punitive escalation.

Third, organizations should critically evaluate the intensity of their formal surveillance systems, recognizing that excessive monitoring may weaken the positive influence of leadership. Our study reveals that ethical leadership is most effective in promoting vigilante behavior when organizational monitoring intensity is low. This suggests that in contexts where strict monitoring is costly or impractical (e.g., remote work or creative teams), ethical leadership becomes an essential substitute for formal control. Conversely, organizations should be cautious about implementing high-intensity monitoring, as it may create a reliance on the system, neutralizing the motivational impact of ethical leaders and reducing employees’ intrinsic motivation to self-regulate and police peers. A more actionable approach is to adopt “calibrated monitoring”: use targeted, risk-based monitoring for high-stakes processes (e.g., compliance-critical tasks) while preserving autonomy in low-risk areas, thereby leaving space for values-based leadership to shape norms. Thus, a shift from control-based management to values-based leadership is recommended for sustainable normative behavior.

### Limitations and future research

First, although this study utilized a multi-source design (i.e., leader–follower matched data) to mitigate common method bias, the cross-sectional nature of the data limits our ability to draw strict causal inferences. Since ethical leadership, team climate, and vigilante behavior are dynamic processes that may change over time, it is difficult to rule out reverse causality. For instance, teams with high levels of vigilante behavior might naturally attract or cultivate more ethical leaders. Future research should adopt longitudinal designs (e.g., cross-lagged panel models) or field experiments to rigorously track these variables over distinct time points, thereby validating the causal direction of the proposed relationships.

Second, the generalizability of our findings may be constrained by the specific cultural and organizational context. Data were collected from a single large enterprise in Central China. Given that organizational vigilantism involves correcting peers, a behavior that is sensitive to cultural norms regarding “face” (mianzi) and harmony in high-context cultures, the mechanisms observed here might differ in Western or individualistic contexts. Future research implies replicating this model across diverse industries and cultural settings. Comparative studies could specifically examine how cultural values (such as power distance or collectivism) interact with organizational monitoring intensity to shape the effectiveness of ethical leadership.

Third, while this study relied on social cognitive theory to identify ethical climate and moral efficacy as key mediators, other theoretical mechanisms may also explain the link between leadership and vigilante behavior. We focused primarily on cognitive and contextual drivers, potentially overlooking affective or relational pathways. Future research could expand the theoretical scope by exploring emotional mechanisms, such as moral anger or moral pride, or relational perspectives, such as Leader-Member Exchange. Additionally, researchers could investigate other boundary conditions beyond organizational monitoring intensity, such as employees’ individual moral identity, to provide a more holistic understanding of when and why employees choose to act as vigilantes.

Fourth, future research should explicitly consider the role of technology in organizational monitoring and its implications for the leadership–vigilantism relationship. Contemporary monitoring is increasingly technology-enabled (e.g., digital trace data, algorithmic surveillance, AI-supported compliance analytics, and remote-work monitoring tools), which may differ qualitatively from traditional human supervision in visibility, perceived intrusiveness, and attribution of control. These technologies may strengthen the “substitute-for-leadership” effect by making oversight more continuous and salient, or alternatively may trigger reactance and privacy concerns that heighten employees’ moral emotions and influence whether vigilantism takes constructive versus punitive forms. Future studies could differentiate types of monitoring technology (human vs. algorithmic; transparent vs. opaque; supportive vs. punitive framing) and test how these features interact with ethical leadership to shape ethical climate, moral efficacy, and subsequent vigilante behavior. Methodologically, combining surveys with digital monitoring metadata (where ethically permissible) may also enable more precise tests of how monitoring intensity and perceived surveillance operate over time.

## Data Availability

The data analyzed in this study is subject to the following licenses/restrictions: the datasets analyzed during the current study are available from the corresponding author upon reasonable request. Due to participant privacy and confidentiality considerations, the data are not publicly posted. Requests to access these datasets should be directed to requests should be sent to Huili Ye, 15872392182@163.com.
